# From the Workroom to the Bedroom: Work-to-Home Spillover as a Mechanism Linking Work Characteristics to Sleep Health

**DOI:** 10.21203/rs.3.rs-4897224/v1

**Published:** 2024-08-13

**Authors:** Kian Huang, Christina Mu, Claire Smith, Soomi Lee

## Abstract

Work may influence the home domain and subsequently impact employee sleep. Past work found that negative spillover mediated the relationship between perceived unfairness about work and insomnia symptoms across 20 years. As an extension of past work, this study investigated whether negative spillover and positive spillover mediate the relationship between job demands (perceived unfairness, job discrimination) and job resources (coworker and supervisor support) on multidimensional sleep health. Two waves of survey data from a subset of full-time workers were obtained from the Midlife in the United States Study approximately 10 years apart. A sleep health composite captured irregularity, dissatisfaction, nap frequency, inefficiency, and suboptimal sleep duration (higher=more sleep health problems). PROCESS Macro evaluated cross-sectional (T1) and sequential (T1 exposureàT1 mediatoràT2 outcome) mediation pathways, adjusting for sociodemographic characteristics, physical health, neuroticism, and work hours. Both cross-sectionally and prospectively, higher negative spillover mediated the association of higher unfairness with more sleep health problems, and the association between higher discrimination and more sleep health problems. There was no support for positive spillover as a mediator between job resources and sleep health cross-sectionally or prospectively. Findings suggest that organizations should reduce the amount of negative spillover by limiting instances of unfairness and discrimination at work to promote specific aspects of employee sleep health such as sleep irregularity, dissatisfaction, efficiency, and nap frequency.

## Introduction

Sleep is imperative for maintaining healthy and robust physical and mental well-being. Insufficient sleep can increase cognitive dysfunction, risk of obesity, cardiovascular morbidity, and depressive symptoms among a myriad of other health consequences (Berryman et al., 2009; Chattu et al., 2019; Haack & Mullington, 2005; Itani et al., 2017; Scott et al., 2021). At work, deficiency in employee sleep relates to increased risks of accidents and injuries, greater fatigue and safety risks, and diminished productivity and cognitive performance (Berryman et al., 2009; Chattu et al., 2019; Colten & Altevogt, 2006). Past research demonstrates that the workplace exposes employees to demands (e.g., perceived unfairness, discrimination) and resources (e.g., social support) that can worsen or improve employee sleep quality and quantity (Lee et al., 2019; Linton et al., 2015; Nakata et al., 2004; Sinokki et al., 2010).

Currently, however, there is little empirical work testing the mechanisms linking work characteristics to employee sleep health, which has stunted the development of strong employee sleep health theories explaining *why* work and sleep are interconnected (Crain et al., 2018). We apply the Work-Home Resources Model (ten Brummelhuis & Bakker, 2012) to test both positive and negative work-to-home spillover – which we will call positive and negative spillover for simplicity – as theoretically supported mechanisms connecting job demands and resources to a variety of important sleep health outcomes (i.e., irregularity, dissatisfaction, nap frequency, inefficiency, and duration). Identifying explanatory mechanisms may also help increase rigor in future intervention studies aiming to understand not only what interventions boost employee sleep health but why (Nielsen & Miraglia, 2016). Further, by expanding upon the limited sleep variables typically included in organizational science research (i.e., quantity and quality; Henderson & Horan, 2021), we shed light onto how employee sleep health, measured more comprehensively, is associated with their work characteristics. Practically, this will also provide information about the holistic extent of these effects across sleep dimensions and specific effects on particular sleep problems. Finally, we address a notable gap in understanding macro-longitudinal patterns in employee sleep research

(Litwiller et al., 2017). Based on previous evidence that the association between work and other health factors differs across cross-sectional and longitudinal study designs (Matthews et al., 2014), we test whether expected links between work characteristics and employee sleep health replicate concurrently and over long-term time (i.e., almost one decade). In total, this study aims to contribute to the mechanistic literature of the work-sleep interface using both negative and positive work-to-home spillover as potential mediators to explain the relationship between work characteristics and multiple key dimensions of employee sleep health.

#### Hypothesis 1

*Consistent with previous literature, the first aim of this study is to establish the relationships between more job demands and poorer sleep health, as well as more job resources and better sleep health.*

### Theoretical Background

To explain the connection between work and home, this study draws upon the Work-Home Resources Model (ten Brummelhuis & Bakker, 2012), which posits that experiences in the work domain can transfer, or *spill over*, to the home domain. Specifically, work-to-home spillover can be defined as the transfer of persisting attitudes, emotions, and behaviors from the work domain to the home domain (Grzywacz & Marks, 2000; Lawson et al., 2013). Work-to-home spillover can take negative or positive forms. *Job demands*, or psychologically straining experiences at work such as unfairness (Lee et al., 2019), may induce negative spillover. On the other hand, *job resources*, or positive work experiences such as perceived coworker and supervisor support may bring about positive spillover (Grzywacz & Marks, 2000; Lawson et al., 2013). In line with this model, several studies have shown that negative spillover mediates the associations of work demands or stressors with personal life outcomes, including relationship quality, well-being, depressive symptoms, family disagreements, chronic health conditions, and satisfaction (Benjamin Goodman et al., 2009; Grzywacz, 2000; Grzywacz & Marks, 2000; Kim et al., 2020; Kinnunen et al., 2006; ten Brummelhuis & Bakker, 2012).

#### Hypothesis 2

*Greater psychological job demands, specifically perceived discrimination and unfairness, will be associated with more negative spillover among workers. Greater job resources, specifically coworker and supervisor support, will be associated with more positive spillover among workers.*

The lack of research concerning the work/sleep interface has created several gaps within the current literature. Past studies have found that interpersonal perceived unfairness and discrimination at work are associated with more sleep problems and insomnia symptoms (Lee et al., 2020; Ohana et al., 2023). Moreover, although limited studies have examined this, research has shown that perceived unfairness about the workplace indirectly increased one specific sleep problem, insomnia symptoms, over time through negative spillover (Lee et al., 2019). Another study showed the mediating effects of negative spillover between job insecurity and subjective sleep quality (Kim et al., 2021).

Additionally, most research concerning the work-sleep interface only focuses on a single measure of sleep, when sleep is in fact multifaceted. Specifically, Buysse’s (2014) Ru-SATED framework is the leading model sleep health in the sleep literature. Ru-SATED encompasses six dimensions of sleep including sleep Regularity (i.e., consistency in sleep timing and/or duration), Satisfaction (i.e., subjective quality), Alertness (i.e., lack of sleepiness during the waking period), Timing (i.e., sleep and wake times), Efficiency (i.e., time to fall asleep and/or time spent awake after sleep period has begun), and Duration (i.e., quantity of sleep; Buysse, 2014; Ravyts et al., 2019). Several studies have highlighted the importance of studying multiple dimensions of sleep (Brindle et al., 2019; Lee et al., 2022). For instance, numerous studies have found that measuring multiple dimensions of sleep (e.g., subjective sleep quality, sleep duration, and sleep efficiency) provided a better understanding of the relationship between sleep and health outcomes than using single-dimension measures (Brindle et al., 2019; Lee et al., 2022; Lee et al., 2021; Wallace et al., 2019) Another study by Lee and Lawson, (2021) found that a multidimensional sleep health composite guided by Ru-SATED had a stronger relationship with psychological and physical well-being compared to the same individual sleep variables independently. Even the meta-analysis by Litwiller et al. (2017), which only examined sleep quantity and quality due to a lack of existing organizational science research on other dimensions, found differential associations between work predictors and sleep depending on the dimension assessed. Clearly, multiple dimensions are involved in healthy sleep, but each may relate differently to job demands and resources.

**Hypothesis 3**

*Negative spillover will mediate the association between job demands and sleep characteristics.*

Even more lacking is the volume of literature analyzing positive work characteristics on sleep health and positive spillover as a mediator between positive work characteristics and sleep outcomes. There is some evidence that greater coworker and supervisor support are associated with higher sleep adequacy (Crain et al., 2014). Similarly, positive spillover is important to study as it is associated with positive health behavior outcomes, including better global sleep quality and quantity (Lee et al., 2014; Williams et al., 2006).

**Hypothesis 4**

*Positive spillover mediates the association between greater coworker and supervisor support and better sleep characteristics.*

### Methods

## Participants and Procedures

Data came from the Midlife in the United States (MIDUS) national study, which was carried out to investigate age-related differences and changes in mental and physical health. Data were collected using phone interviews and self-assessment questionnaires. The first wave of data collection was between 1995–1997. Additional follow-ups occurred between 2004–2006 (MIDUS 2; $n$ = 5,555) and 2013–2015 (MIDUS 3; $n$ = 3,683), and included more comprehensive sleep and work measures that were analyzed in this study. Reasons for attrition include changes in eligibility (e.g., participant became cognitively impaired), death, loss of contact, and non-compliance (Brim et al., 2004). In this paper, MIDUS 2 will be referred to as Time 1 (T1) and MIDUS 3 will be referred to as Time 2 (T2). Details regarding the procedure and design of the MIDUS study are published elsewhere (Brim et al., 2004; Radler, 2014). Data are publicly available: http://www.midus.wisc.edu/.

Given that we were interested in work characteristics and work-to-home spillover, we restricted our sample to full-time workers (working 30 hours or more per week as defined by the Affordable Care Act; IRS, 2023). Of the 5,555 participants at T1, 3,009 were full-time workers. Of the full-time workers, 909 participants had missingness on work characteristics ($n$ = 816), work-to-home spillover ($n$ = 8), sleep ($n$ = 67), or sociodemographic characteristics

(*n* = 18). Thus, our final sample for the cross-sectional analyses at T1 were between 1,801-2,100 participants per model; models varied due to different missingness on work characteristics. At T2 follow-up, 1,444 participants provided valid sleep, spillover, and work characteristics.

The larger MIDUS study was approved by the University of Wisconsin-Madison Institutional Review Board (IRB). MIDUS participants provided written informed consent. The current study was exempt from IRB review because it used secondary, de-identifiable data.

## Measures

## Job Demands

All variables were measured consistently at T1 and T2. *Job demands* were captured by perceived unfairness and discrimination. *Job resources* were captured by coworker and supervisor support.

**Perceived Unfairness.** Participants rated the level of perceived unfairness about work using a 6-item scale (Ryff et al., 2003). The items read: "(1) I feel cheated about the chances I have had to work at good jobs, (2) When I think about the work I do on my job, I feel a good deal of pride, (3) I feel that others respect the work I do on my job, (4) Most people have more rewarding jobs than I do, (5) When it comes to my work life, I've had opportunities that are as good as most people's (reverse coded), and (6) It makes me discouraged that other people have much better jobs that I do." Responses options were 1 (*A lot*), 2 (*Some*), 3 (*A little*), and 4 (*Not at all*). The response scale and certain items were reverse coded, so higher values indicated more perceived unfairness. Cronbach's alpha was .75 at T1 and .75 at T2.

**Job Discrimination**. Participants rated the level of perceived job discrimination at work based on a 6-item scale (Brim et al., 2004). The items read: "(1) How often do you think you are unfairly given the jobs that no one else wanted to do?, (2) How often are you watched more closely than other workers?, (3) How often does your supervisor or boss use ethnic, racial, or sexual slurs or jokes?, (4) How often do your coworkers use ethnic, racial, or sexual slurs or jokes?, (5) How often do you feel that you are ignored or not taken seriously by your boss?, (6) How often has a coworker with less experience and qualifications gotten promoted before you?" (Ryff et al., 2004). Response options were 1 (*Never*), 2 (L*ess than once a year*), 3 (*A few times a year*), 4 (*A few times a month*), and 5 (*Once a week or more*). Higher values indicated more job discrimination. Cronbach's alpha was .78 at T1 and .79 at T2.

**Coworker Support**. Participants rated the level of coworker support at work based on a 2-item scale previously used in other studies (Ettner & Grzywacz, 2001; Grzywacz & Marks, 2000). The items read: "(1) How often do you get help and support from your coworkers? and (2) How often are your coworkers willing to listen to your work-related problems?" (Brim et al., 2004). Each question was coded as 1 (*All of the time*), 2 (*Most of the time*), 3 (*Sometimes*), 4 (*Rarely*), and 5 (*Never*). All items were reverse-coded, so that higher values indicated more coworker support. Cronbach's alpha was .67 at T1 and .72 at T2.

**Supervisor Support**. Participants rated the level of supervisor support at work based on a 3-item scale previously used (Ettner & Grzywacz, 2001; Grzywacz & Marks, 2000). The items read: "(1) How often do you get the information you need from your supervisor or superiors?", (2) "How often do you get help and support from your immediate supervisor?", (3) "How often is your immediate supervisor willing to listen to your work-related problems?" Each question was coded as 1 (*All of the time*), 2 (*Most of the time*), 3 (*Sometimes*), 4 (*Rarely*), and 5

(*Never*). All items were reverse-coded, so that higher values indicated more supervisor support. Cronbach's alpha was .87 at T1 and .87 at T2.

**Negative Spillover.** Participants rated the level of negative spillover using a 4-item scale (Grzywacz, 2000; Grzywacz & Marks, 2000). The items read: "(1) Your job reduces the effort you can give to activities at home, (2) Stress at work makes you irritable at home, (3) Your job makes you feel too tired to do the things that need attention at home, (4) Job worries or problems distract you when you are at home." Each question was coded as 1 (*All of the time*), 2 (*Most of the time*), 3 (*Sometimes*), 4 (*Rarely*), and 5 (*Never*). All items were reverse-coded, so higher values indicated a higher level of negative spillover. Cronbach's alpha was .82 at T1 and .85 at T2.

**Positive Spillover.** positive spillover was assessed using a 4-item scale (Grzywacz, 2000; Grzywacz & Marks, 2000). The items read: "(1) The things you do at work help you deal with personal and practical issues at home?, (2) The things you do at work make you a more interesting person at home, (3) Having a good day on your job makes you a better companion when you get home, and (4) The skills you use on your job are useful for things you have to do at home." Each question was coded as 1 (*All of the time*), 2 (*Most of the time*), 3 (*Sometimes*), 4 (*Rarely*), and 5 (*Never*). All items were reverse-coded, so higher values indicated a higher level of positive spillover. Cronbach's alpha was .72 at T1 and .73 at T2.

**Sleep Health Problems**. Sleep health problems were captured in two ways: (1) using individual dimension scores and (2) using a composite of 5 dimensions in Buysse's (2014) Ru-SATED model. The model captured sleep irregularity, dissatisfaction, nap frequency (alertness), inefficiency, and suboptimal sleep duration, but could not capture timing because this information was not available in the MIDUS survey data. We created binary variables for each sleep dimension following cut-off points used in the previous literature (Lee et al., 2022; Smith et al., 2023), such that 0 represents good sleep and 1 represents poor sleep or a sleep problem.

*Irregularity* was defined as the absolute value difference in sleep duration between workdays and nonwork days. Irregularity was expressed as a binary variable where the absolute value difference of more than 60 minutes was coded as 1 and 60 minutes or less was coded as 0. *Dissatisfaction* was assessed by 4 insomnia symptoms following previous literature (Lee et al., 2022; Knutson et al., 2017): (1) Having trouble falling asleep, (2) waking up during the night, and having trouble falling back to sleep, (3) waking up early in the morning, and having trouble falling back to sleep, and (4) feeling unrested throughout the daily regardless of the duration of last night's sleep. Respondents answered each item as either 1 (sometimes, often, or almost always) or 0 (rarely or never) on each item. The dissatisfaction variable was dichotomized by assigning 0 to responses with "Rarely or Never" on all 4 items and assigning 1 to responses with at least one "Sometimes, Often, or Almost Always" response to 1 of the 4 items. *Nap frequency (lack of alertness)* was operationalized as the number of times one napped for 5 minutes or more during the week. Nap frequency was expressed as a binary variable where more than 2 naps a week was coded as 1 and 2 naps or less a week was coded as 0. MIDUS does not capture sleep timing, so this dimension of Ru-SATED was excluded. *Inefficiency* was defined as how long it took the respondent to fall asleep. Inefficiency was measured by sleep onset latency. We created a binary indicator with 1 equal to respondents taking more than 30 minutes to fall asleep, and 0 equal to respondents taking 30 minutes or less to fall asleep. Lastly, suboptimal sleep *duration* was captured by the average amount of sleep the participant received on workdays. Average sleep durations that were less than 6 hours or greater than 8 hours were coded as 1 and reported sleep between 6 to 8 hours was coded as 0. To create the composite score, all binary indicators were summed such that possible scores ranged from 0 to 5, with higher scores representing more sleep health problems.

## Covariates

Fully adjusted models controlled for potentially confounding variables, which have been shown to be associated with sleep and work characteristics (Adults et al., 2012; Grandner et al., 2010). Covariates were age (in years), sex (1 = Men, 0 = Women), race (0 = Non-Hispanic White, 1 = Hispanic Whites/Person of color), education (1 = no school/some grade school to 12 = professional degrees such as Ph.D., ED.D., or MD), partnered status (0 = partnered including married and cohabitating, 1 = single including un married, separated, and divorced), the number of children living in the household, self-rated physical health (1 = Poor to 5 = Excellent), and average amount of work hours per week. We also controlled for neuroticism (1 = Less neurotic to 4 = More neurotic), as it is a known predictor of poor sleep health and may influence the perception and response to questions on workplace experiences and spillover (Duggan et al., 2014; Widiger & Oltmanns, 2017). All continuous variables were centered at the sample means. For the longitudinal analyses, all covariates, except race and sex, were measured at T2. Following Lee and colleagues (2019), we controlled for changes in partnered status over time; 4 categories were created so that those who were continuously partnered (married/cohabitating at both T1 and T2) were coded 1, single to partnered (e.g., unmarried at T1 and married at T2) were coded 2, single throughout (e.g., unmarried at both T1 and T2) were coded 3, or other change patterns (e.g., married at T1 and divorced at T2) were coded 4.

## Statistical Analyses

We tested the study hypotheses cross-sectionally and prospectively. The cross-sectional analyses used data from T1, and prospective analyses used work characteristics and spillover from T1 and sleep health from T2 (see Fig. 1). For both approaches, analyses were conducted separately for each work characteristic and for positive spillover or negative spillover. Fully adjusted general linear models were first used to examine individual paths (a, b, c, and c'). Indirect effect tests were tested using PROCESS Macro (Hayes, 2017; Preacher & Hayes, 2004). All analyses were conducted in SAS v9.4 (SAS Institute, Cary NC). Statistical significance was determined if the 95% confidence interval did not include 0.

Supplemental analyses were conducted to examine 1) the cross-sectional associations between work characteristics and individual sleep dimensions, and 2) change score analyses with work characteristics from T1, the change in spillover from T1 to T2, and sleep health at T2. To test the individual sleep dimensions, we examined the continuous variables for sleep irregularity (absolute value of the difference between workday and non-workday sleep), sleep dissatisfaction (count variable of number of insomnia symptoms, Range = 0–4), average nap frequency over the week (Range = 0–12), sleep latency (time in decimal hours), and average sleep duration (time in decimal hours).

### Results

Table 1 displays the sample characteristics. At baseline, the average age of the participants was 50.0 years (*SD* = 8.8), and worked an average of 44.9 hours per week (*SD* = 9.7). About half (52.1%) were men, and 84.5% were non-Hispanic White. The final level of education was 7.5 (SD = 2.5), which corresponds to having graduated from a 2-year college, vocational school, or completion of an Associate's degree. The majority of participants were married or partnered (70.3%). The average number of children in the participant household was 2 (SD = 1.7). Participants' self-rated physical health (1 = Excellent to 5 = Poor) was 2.3 (*SD* = 0.89), which was close to "very good." The average level of neuroticism (1–4 scale) was fair (*M* = 2.1, *SD* = 0.6). Characteristics were mostly retained at T2, except age and partnered status in the prospective sample was older and included fewer partnered people. See Supplemental Table 1 for correlations between study variables.

Table 1
*Descriptive statistics of study variables at baseline.*

| | **Baseline** (***n*** **= 2,100)** |
|---|---|
| Main Variables | |
| Unfairness/Perceived Inequality (Range = 1–4), *M* (SD) | 1.60 (0.54) |
| Chronic Job Discrimination (Range = 6–30), *M* (SD) | 11.10 (4.44) |
| Coworker Support (Range = 2–10), *M* (SD) | 7.22 (1.47) |
| Supervisor Support (Range = 3–15), *M* (SD) | 10.70 (2.64) |
| Sleep Problems (Range = 0–5), *M* (SD)[a] | 1.59 (1.06) |
| Mediators[a] | |
| Positive Work-to-home Spillover (Range = 4–20), *M* (SD) | 11.68 (2.81) |
| Negative Work-to-home Spillover (Range = 4–20), *M* (SD) | 10.44 (2.65) |
| Covariates | |
| Age (in years), *M* (SD) | 49.97 (8.84) |
| Work hours, *M* (SD) | 44.89 (9.72) |
| Sex, *n* (%) | |
| Men | 1095 (52.14%) |
| Women | 1005 (47.86%) |
| Race, *n* (%) | |
| Non-Hispanic White | 1774 (84.48%) |
| Person of Color | 326 (15.52%) |
| Highest education level (1–12)[b], *M* (SD) | 7.52 (2.49) |
| Married/partnered, *n* (%)[c] | 1476 (70.29%) |
| Children in household, *M* (SD) | 2.26 (1.66) |
| Self-rated physical health (1:Excellent – 5:Poor), *M* (SD) | 2.25 (0.89) |
| Neuroticism (1–4), *M* (SD) | 2.10 (0.62) |

*Note.* [a]Sleep health at T2 was *M*(*SD*) = 1.68(1.07).[b]Education was coded as 1 = no school/some grade school to 12 = professional degrees (e.g., Ph.D., ED.D., MD). Partnered status was collected at baseline as either married/partnered or singled Partnered status 10 years later (T2) was categorized as either married throughout (married at both T1 and T2), single to partnered (unmarried at T1 and married at T2), single throughout (unmarried at both T1 and T2), or other change patterns (married at T1 and unmarried at T2 etc.).

## Cross-Sectional Results

Table 2 and Fig. 2 display results from the cross-sectional associations, organized into the respective paths (a, b, c, and c’). Hypothesis 1, which tested the associations between work characteristics and sleep problems (c path), was partially supported. Higher perceived unfairness ($B$ = 0.14, 95% CI [0.06 to 0.22]) and higher chronic job discrimination ($B$ = 0.03, 95% CI [0.02 to 0.03]) were positively associated with more sleep health problems. In terms of job resources, neither coworker support nor supervisor support were associated with sleep health problems.

Table 2

*Cross-sectional associations between work characteristics and overall sleep health mediated by work-to-home spillover at T1.*

| **A and B paths** | ***n*** | ***B*** | **SE** | **95% Lower CI** | **95% Upper CI** | **Effect** | **Boot SE** | **Boot 95% Lower CI** | **Boot 95% Upper CI** |
|---|---|---|---|---|---|---|---|---|---|
| Perceived Unfairness → Negative Spillover | 2552 | **0.95** | **0.09** | **0.77** | **1.14** | | | | |
| Negative Spillover → Sleep Problems | | **0.03** | **0.01** | **0.02** | **0.05** | | | | |
| Chronic Job Discrimination → Negative Spillover | 2547 | **0.18** | **0.01** | **0.15** | **0.20** | | | | |
| Negative Spillover → Sleep Problems | | **0.03** | **0.01** | **0.01** | **0.04** | | | | |
| Coworker Support → Positive Spillover | 2287 | **0.40** | **0.04** | **0.32** | **0.47** | | | | |
| Positive Spillover → Sleep Problems | | -0.01 | 0.01 | -0.03 | 0.002 | | | | |
| Supervisor Support → Positive Spillover | 2144 | **0.20** | **0.02** | **0.15** | **0.24** | | | | |
| Positive Spillover → Sleep Problems | | -0.01 | 0.01 | -0.03 | 0.002 | | | | |
| **C path (Total Effect)** | **n** | **B** | **SE** | **95% Lower CI** | **95% Upper CI** | | | | |
| Perceived Unfairness → Sleep Problems | 2552 | **0.14** | **0.04** | **0.06** | **0.22** | | | | |
| Chronic Job Discrimination → Sleep Problems | 2547 | **0.03** | **0.005** | **0.02** | **0.03** | | | | |
| Coworker Support → Sleep Problems | 2287 | -0.02 | 0.01 | -0.05 | 0.01 | | | | |
| Supervisor Support → Sleep Problems | 2144 | -0.01 | 0.01 | -0.03 | 0.004 | | | | |
| **C' path (Direct Effect)** | **n** | **B** | **SE** | **95% Lower CI** | **95% Upper CI** | **Effect** | **Boot SE** | **Boot 95% Lower CI** | **Boot 95% Upper CI** |
| Perceived Unfairness + Negative Spillover → Sleep Problems | 2552 | **0.11** | **0.04** | **0.03** | **0.19** | **0.03** | **0.009** | **0.014** | **0.049** |

*Note*. Sample sizes vary per model due to differences in missingness in work characteristics. Significant models are bolded.

| A and B paths | *n* | *B* | SE | 95% Lower CI | 95% Upper CI | Effect | Boot SE | Boot 95% Lower CI | Boot 95% Upper CI |
|---|---|---|---|---|---|---|---|---|---|
| Chronic Job Discrimination + Negative Spillover → Sleep Problems | 2547 | **0.02** | **0.01** | **0.01** | **0.03** | **0.005** | **0.002** | **0.002** | **0.008** |
| Coworker Support + Positive Spillover → Sleep Problems | 2287 | -0.02 | 0.01 | -0.04 | 0.01 | -0.005 | 0.003 | -0.012 | 0.001 |
| Supervisor Support + Positive Spillover → Sleep Problems | 2144 | -0.01 | 0.01 | -0.03 | 0.01 | -0.003 | 0.002 | -0.006 | 0.0004 |
| *Note*. Sample sizes vary per model due to differences in missingness in work characteristics. Significant models are bolded. | | | | | | | | | |

Hypothesis 2 examined the association between work characteristics and spillover (a path). Higher perceived unfairness (*B* = 0.95, 95% CI [0.77 to 1.14]) and chronic job discrimination (*B* = 0.18, 95% CI [0.15 to 0.20]) were associated with higher negative spillover. Turning to job resources, higher coworker support (*B* = 0.40, 95% CI [0.32, 0.47]) and supervisor support (*B* = 0.20, 95% CI [0.15, 0.24]) were also associated with higher positive spillover.

Negative spillover mediated the relationships between higher perceived unfairness and poorer sleep health (indirect effects: *B* = 0.03, 95% CI [0.014, 0.049]) and higher job discrimination and poorer sleep health (indirect effects: *B* = 0.005, 95% CI [0.002, 0.008]), which supported Hypothesis 3. Hypothesis 4 was not supported; in that, positive spillover did not mediate the relationships between more coworker support (indirect effects: *B*=-0.005, 95% CI [-0.012, 0.001]) and supervisor support (indirect effects: *B*=-0.003, 95% CI [-0.006, 0.0004]) on overall sleep health.

## Prospective Results

Table 3 and Fig. 3 display results from prospective models, organized into the respective paths (a, b, c, and c'). For Hypothesis 1, there was support for negative but not positive work characteristics. Both higher perceived unfairness (*B* = 0.16, 95% CI [0.06, 0.26]) and higher job discrimination (*B* = 0.02, 95% CI [0.01, 0.04]) at T1 were associated with poorer sleep health at T2. Higher job resources at T1 were not associated with better sleep health at T2.

Table 3

*Prospective relationships between work characteristics at T1 and overall sleep health at T2 mediated by work-to-home spillover from T1.*

| **A and B paths** | ***n*** | ***B*** | **SE** | **95% Lower CI** | **95% Upper CI** | **Indirect Effects Tests** | | | |
|---|---|---|---|---|---|---|---|---|---|
| Perceived Unfairness → Negative Spillover | 1850 | **0.89** | **0.11** | **0.67** | **1.11** | | | | |
| Negative Spillover → Sleep Problems | | **0.03** | **0.01** | **0.01** | **0.05** | | | | |
| Chronic Job Discrimination → Negative Spillover | 1844 | **0.16** | **0.01** | **0.14** | **0.19** | | | | |
| Negative Spillover → Sleep Problems | | **0.03** | **0.01** | **0.01** | **0.05** | | | | |
| Coworker Support → Positive Spillover | 1691 | **0.36** | **0.04** | **0.28** | **0.45** | | | | |
| Positive Spillover → Sleep Problems | | -0.01 | 0.01 | -0.03 | 0.01 | | | | |
| Supervisor Support → Positive Spillover | 1564 | **0.19** | **0.03** | **0.14** | **0.24** | | | | |
| Positive Spillover → Sleep Problems | | -0.01 | 0.01 | -0.03 | 0.01 | | | | |
| **C path (Total Effect)** | **n** | **B** | **SE** | **95% Lower CI** | **95% Upper CI** | | | | |
| Perceived Unfairness → Sleep Problems | 1850 | **0.16** | **0.05** | **0.06** | **0.26** | | | | |
| Chronic Job Discrimination → Sleep Problems | 1844 | **0.02** | **0.01** | **0.01** | **0.04** | | | | |
| Coworker Support → Sleep Problems | 1691 | -0.03 | 0.02 | -0.06 | 0.01 | | | | |
| Supervisor Support → Sleep Problems | 1564 | -0.01 | 0.01 | -0.03 | 0.01 | | | | |
| **C' path (Direct Effect)** | **n** | **B** | **SE** | **95% Lower CI** | **95% Upper CI** | **Effect** | **Boot SE** | **Boot 95% Lower CI** | **Boot 95% Upper CI** |
| Perceived Unfairness + Negative Spillover → Sleep Problems | 1850 | **0.13** | **0.05** | **0.03** | **0.23** | **0.030** | **0.011** | **0.010** | **0.052** |
| Chronic Job Discrimination + | 1844 | **0.02** | **0.01** | **0.01** | **0.03** | **0.005** | **0.002** | **0.001** | **0.009** |

*Note*. Sample sizes vary per model due to differences in missingness in work characteristics.

| A and B paths | *n* | *B* | SE | 95% Lower CI | 95% Upper CI | Indirect Effects Tests | | | |
|---|---|---|---|---|---|---|---|---|---|
| Negative Spillover → Sleep Problems | | | | | | | | | |
| Coworker Support + Positive Spillover → Sleep Problems | 1691 | -0.02 | 0.02 | -0.06 | 0.01 | -0.003 | 0.004 | -0.011 | 0.004 |
| Supervisor Support + Positive Spillover → Sleep Problems | 1564 | -0.01 | 0.01 | -0.03 | 0.01 | -0.001 | 0.002 | -0.005 | 0.003 |
| *Note*. Sample sizes vary per model due to differences in missingness in work characteristics. | | | | | | | | | |

Hypothesis, which examined the associations between spillover and sleep health at T1, remained significant among those who provided T2 sleep data. That is, more negative work characteristics were associated with negative spillover, and more positive work characteristics were associated with positive spillover.

Negative spillover at T1 mediated the relationship between higher perceived unfairness at T1 and poorer sleep health at T2 (indirect effects: *B* = 0.03, 95% CI [0.010, 0.052]), and the association between higher job discrimination at T1 and poorer sleep health at T2 (indirect effects: *B* = 0.005, 95% CI [0.001, 0.009])—supporting Hypothesis 3. Contrastingly, hypothesis 4, that higher positive spillover at T1 mediated the relationship between greater job resources at T1 and better sleep health at T2, was not supported. Positive spillover at T1 did not mediate the relationship between coworker support at T1 and sleep health at T2 (indirect effects: *B*=-0.003, 95% CI [-0.011, 0.004]) or supervisor support at T1 and sleep health at T2 (indirect effects: *B*=-0.001, 95% CI [-0.005, 0.003]).

## Supplemental Analyses

Supplemental Table 2 displays the cross-sectional analyses with the individual sleep dimensions (i.e., irregularity, dissatisfaction, nap frequency, inefficiency, and duration) to understand which dimensions of employee sleep health were more sensitive to work characteristics and work-to-home spillover. Each sleep dimension was tested in a separate model. Higher perceived unfairness was associated with more irregular sleep (*B* = 0.11, 95% CI [0.03, 0.18]), sleep dissatisfaction (*B* = 0.23, 95% CI [0.13, 0.33]), and lack of alertness (*B* = 0.21, 95% CI [0.04, 0.38]). Higher chronic job discrimination was associated with more irregular sleep (*B* = 0.02, 95% CI [0.01, 0.03]) and sleep dissatisfaction (*B* = 0.03, 95% CI [0.02, 0.05]). Higher coworker support was associated with lower sleep dissatisfaction (*B*=-0.05, 95% CI [-0.08, -0.01]) and longer sleep duration (*B* = 0.04, 95% CI [0.01, 0.07]). Higher supervisor support was associated with lower sleep dissatisfaction (*B*=-0.03, 95% CI [-0.05, -0.01]).

Higher negative spillover at T1 mediated the relationships between perceived unfairness at T1 on sleep irregularity (indirect effect: *B* = 0.02, 95% CI [0.01, 0.05]) and sleep dissatisfaction at T2 (indirect effect: *B* = 0.07, 95% CI [0.05, 0.10]), as well as the relationships between chronic job discrimination at T1 and sleep irregularity at T2 (indirect effect: *B* = 0.004, 95% CI [0.0001, 0.01]) and sleep dissatisfaction at T2 (indirect effect: *B* = 0.01, 95% CI [0.01, 0.02]). Positive spillover did not mediate any of the associations between positive work characteristics and sleep dimensions.

Supplemental Table 3 displays the prospective analyses with the individual sleep dimensions at T2. Prospectively, higher perceived unfairness was associated with more irregular sleep (*B* = 0.09, 95% CI [0.01, 0.18]), higher sleep dissatisfaction (*B* = 0.23, 95% CI [0.11, 0.36]), and lower sleep efficiency (*B* = 0.06, 95% CI [0.01, 0.10]). Similarly, chronic job discrimination was associated with more irregular sleep (*B* = 0.02, 95% CI [0.01, 0.03]), higher sleep dissatisfaction (*B* = 0.03, 95% CI [0.02, 0.05]), lower sleep efficiency (*B* = 0.01, 95% CI [0.01, 0.02]), and shorter sleep duration (*B*=-0.02, 95% CI [-0.03, -0.01]) at T2. Higher negative spillover at T1 mediated the associations between perceived unfairness on sleep dissatisfaction (indirect effect: *B* = 0.06, 95% CI [0.03, 0.09]) and lack of alertness (indirect effect: *B* = 0.05, 95% CI [0.02, 0.10]). Higher negative spillover at T1 mediated the associations between chronic job discrimination at T1 and sleep dissatisfaction at T2 (indirect effect: *B* = 0.01, 95% CI [0.007, 0.02]) and lack of alertness (indirect effect: *B* = 0.01, 95% CI [0.004, 0.02]). Positive spillover did not mediate any of the cross-sectional prospective results between work characteristics and individual sleep dimensions.

Additional supplemental analyses were conducted to examine the mediating effects of change in spillover from T1 to T2 on the relationship between work characteristics at T1 and sleep problems at T2 (Supplemental Table 4). Results revealed that changes in work-to-family spillover from T1 to T2 did not mediate the relationship between work characteristics at T1 and sleep problems at T2.

## Discussion

Guided by the Work-Home Resources Model (Ten Brummelhuis & Bakker, 2012), this paper investigated the associations of job demands and resources with multidimensional sleep health via negative and positive work-to-home spillover, respectively. Negative spillover consistently emerged as an explanatory mediator linking job demands with poorer sleep health both cross-sectionally and prospectively 10 years later. In supplementary analyses, negative spillover also mediated the relationship between job demands and sleep irregularity, dissatisfaction, and lack of alertness. Unexpectedly, though, positive spillover was not a significant mediator between job resources and poorer sleep health. In fact, job resources were not significantly associated with total sleep health cross-sectionally or prospectively, though a few dimensional results were significant cross-sectionally (i.e., more coworker and supervisor were associated with higher positive spillover, sleep satisfaction, and longer sleep duration). Our findings show that negative spillover explains how job demands negatively influence employee sleep health. These findings yield important points to discuss, for both research and practice.

A novel contribution of the present study is the exploration of positive work characteristics and positive spillover. Previous studies found associations between more job resources and higher positive spillover and higher positive spillover with better sleep health (Beham et al., 2023; Williams et al., 2006; Zayed et al., 2021). Results of this study revealed that job resources were positively associated with positive spillover, yet neither job resources nor positive spillover had a direct association with sleep health problems. In spite of the null findings with positive spillover as a mediator between the job resources and sleep health relation, these results provide new insight into the unique nature of the psychological effects negative experiences have on humans. Negative events have a stronger influence on health compared to positive events (Baumeister et al., 2001), due to the negativity bias wherein people tend to pay more attention to negative information, have stronger emotional responses to negative events, and remember negative experiences more vividly and for a more extended period than positive ones (Baumeister et al., 2001). Our stronger support for negative spillover of stressful work experiences to sleep health, compared to positive spillover of supportive work experiences, fit within this broader theme. Both theoretically and practically, protecting sleep health may be a function of limiting job demands, though boosting job resources unfortunately may not have substantial, long-term benefits for actively promoting healthier sleep.

Another important contribution of this study is our approach to sleep health as multidimensional. Using a wider variety of sleep health dimensions, our research brings the use of sleep health composites to an organizational science audience, following evidence of their influence above and beyond individual dimensions in the broader sleep literature (Lee et al., 2019). Clearly, work experiences, particularly demands, can spill over to relate to a wider array of sleep problems than previously examined. Further, by examining this more comprehensive constellation of sleep health facets dimensionally in our supplemental analyses, we expand upon previous findings that job demands generally relate to more sleep health problems (Lee et al., 2019; Lee et al., 2020). Our supplemental results point to two specific sleep dimensions (i.e., irregularity, dissatisfaction) that may be more sensitive to concurrent negative and positive experiences at work. This finding both complements and expands upon traditional focus on sleep duration (quantity) and satisfaction (quality) in previous organizational science research (Litwiller et al., 2017). As a complement, our results agree with past work that satisfaction or sleep quality exhibits stronger and more consistent associations with work factors, relative to sleep duration (Barber et al., 2017; Litwiller et al., 2017). However, we also add sleep schedule regularity as a prominent sleep health variable that may be affected by work experiences. Regularity has been occasionally discussed in previous research (Barber et al., 2010) but remains largely overlooked relative to quantity and quality. Certain sleep variables, such as sleep satisfaction and regularity, uniquely have more persisting negative effects than others, calling for the need for a multivariate approach to measuring sleep health outcomes as existing studies are also limited to a single measure of sleep. Future research should base their sleep measurement decisions on whether broad sleep health or specific sleep issues are more theoretically and/or practically relevant to the particular research question, since both appear to be meaningfully related to demands and resources at work.

## Practical Implications

Our findings offer real-world implications for managing overlooked aspects of employee sleep health, including over the long-term. Of these measures, perceived unfairness and job discrimination appeared to have the strongest and most consistent effects on overall employee sleep health, pointing to it as a key target for organizational sleep health interventions. Although, other important aspects of sleep, such as sleep irregularity, may serve as new targets for workplace interventions to address sleep health problems in employees. Relevant to addressing perceived unfairness as a demand, previous occupational literature finds that participative interventions – where employees get to contribute their input into the decisions and organization of the workplace – tend to increase equity and inclusion and, thus, may be effective when trying to create a fair workplace that supports employee health and well-being (Linna et al., 2011). Workplaces can use strategies at both the individual and organizational level to reduce job discrimination such as promoting advocacy and allyship and integrating evidence-based diversity training targeted towards implicit bias and prejudice (Hebl et al., 2020). Overall, businesses and organizations should consider how leadership may enforce anti-discriminatory policies and address employee complaints and concerns as a method to effectively deter discrimination within the workplace (Larsen et al., 2013).

Further, our results show that negative spillover is a consistent mechanism linking job demands to sleep health and, therefore, a useful proximal target for intervention when work conditions cannot be changed sufficiently. Previously identified strategies, such as practicing gratitude when reflecting on a day’s work and detaching from work during non-work hours (Sonnentag et al., 2020), can reduce negative spillover and subsequently the harmful effects of negative work characteristics on health.

## Limitations and Future Directions

The limitations of this study may serve as helpful context in interpreting results and further guidance for future research and discussion. First, the sample was majority non-Hispanic White (85%) which, although only slightly higher than the U.S. population (73%) still does not allow for more targeted examination of the association between work and sleep for people of color -- who, on average, experience disproportionately more job demands such as discrimination as well as poorer sleep health (Fekedulegn et al., 2019; Wens et al., 2017). Second, seeing as both the predictor and criterion variables were recorded from a self-reported survey, common method bias could obscure the true relationships (Podsakoff et al., 2003). That said, temporal separation of the baseline and follow-up surveys may reduce common method bias concerns for the prospective analyses. Future directions of this study are to use objective sleep health measures, (e.g., actigraphy), rather than self-reported survey data. Third, for the cross-sectional prospective and prospective analyses, only two waves of data were used, separated by a 10-year lag. Incorporating three or more waves allows for a time lag between each supposed cause and effect in mediation models, though these designs do not provide the clear-cut, consistently reliable solution that they are sometimes assumed to (Reichardt, 2011). Using a full longitudinal design and employing different timeframes (e.g., over weeks, months, or additional years) will help fully understand the temporal relationships between variables.

## Conclusion

Interpersonal job demands (i.e., workplace unfairness and discrimination) were associated with more employee sleep health problems, whereas job resources (i.e., coworker and supervisor support) were not associated with sleep health problems. Negative spillover acted as a mechanism for the associations between job demands and poorer sleep health, but positive spillover did not mediate associations between job resources and better sleep health. For perceived unfairness, the effect was so pronounced that it even persisted 10 years later in its association with higher employee sleep irregularity, dissatisfaction, and lack of alertness. Similarly, higher job discrimination was associated with higher employee sleep irregularity and dissatisfaction 10 years later. These findings point to specific avenues to intervene (i.e., job discrimination and negative work-to-home spillover) that are important to maintaining employees' comprehensive sleep health.

## Declarations

**Author Contributions**: KH initiated this project, conducted the analyses, and drafted the manuscript. CM and CS supervised the data management and analyses. All authors contributed to the conceptualization and revision of this manuscript, as well as approved the final version.

**Conflicts of Interests**: None.

**Funding**: Outside of the current work, Dr. Soomi Lee discloses that she received funding support from NIH/National Institute on Aging (Grant No. R56AG065251) and NIH/National Heart, Lung, and Blood Institute (Grant No. R01HL163226, PI: Lee). Christina Mu discloses that she received funding support from NIH/National Heart, Lung, and Blood Institute (Grant No. 1F31HL165898-01, PI: Mu). The content is solely the responsibility of the authors and does not represent the official views of the NIH.

## Figures

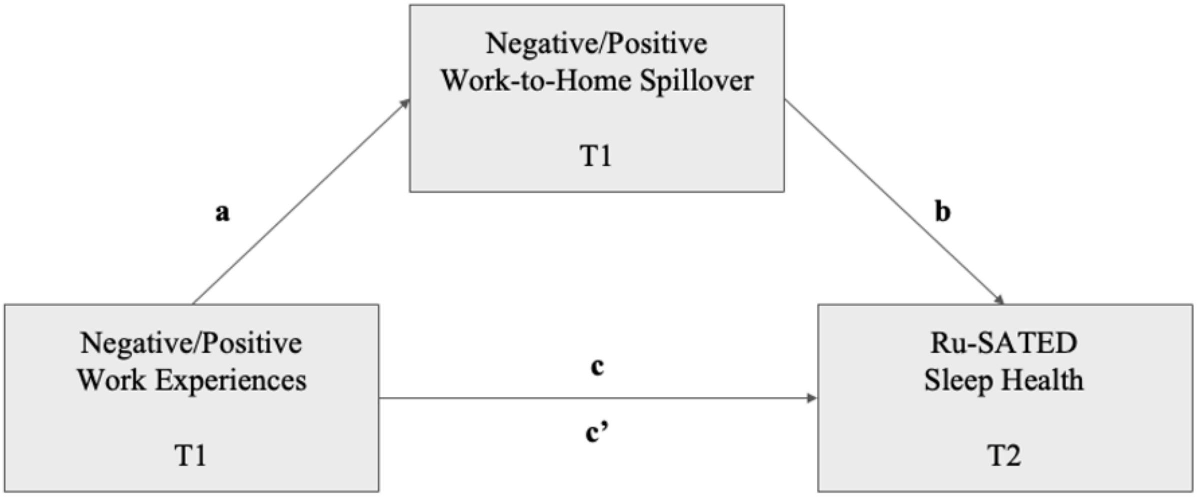

### Figure 1

*Research model illustrating the cross-sectional prospective associations of work characteristics and multidimensional sleep health, mediated by Negative Spillover and Positive Spillover.*

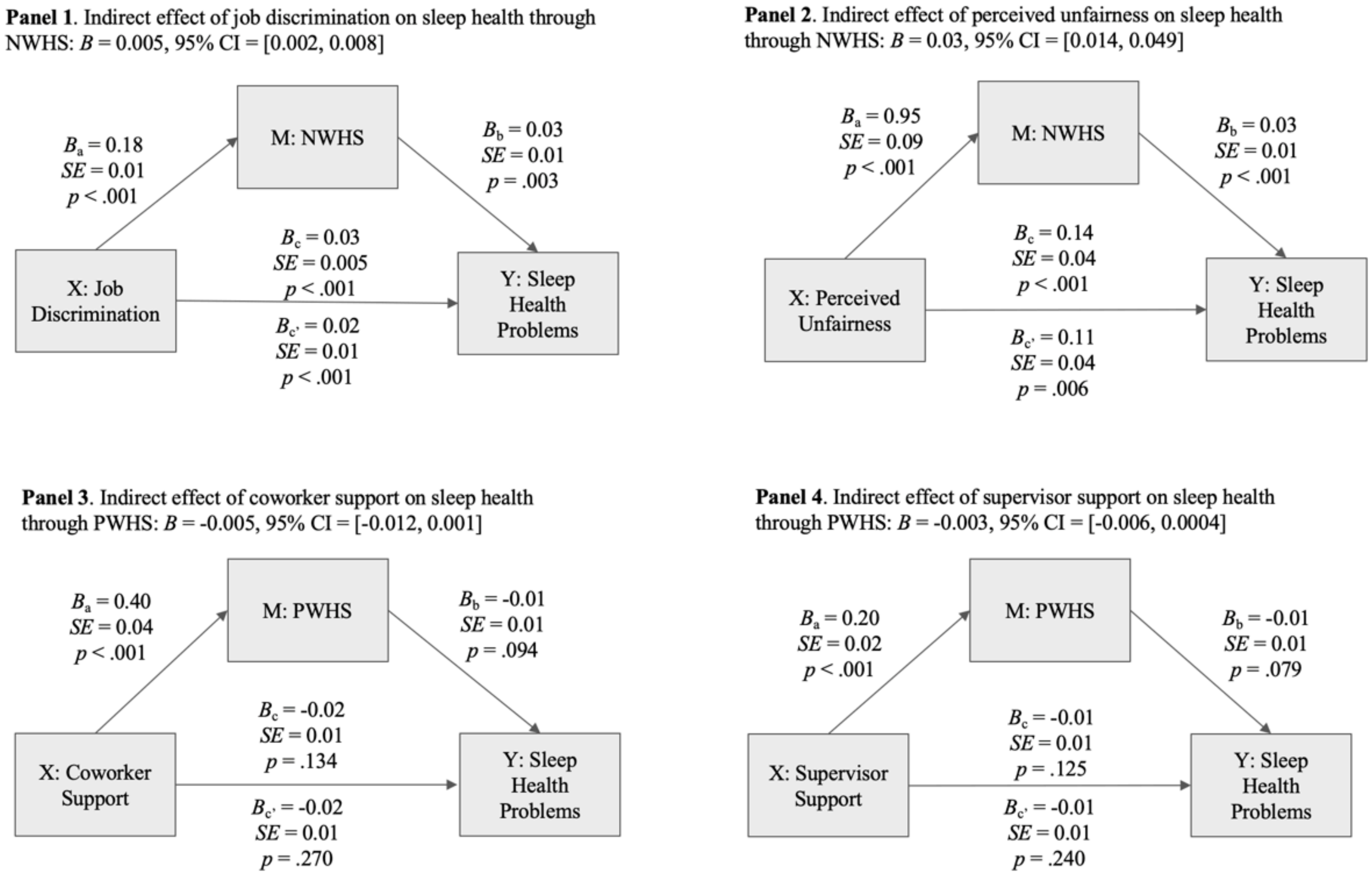

### Figure 2

*Cross-sectional associations between work characteristics and overall sleep health mediated by work-to-home spillover at T1.*

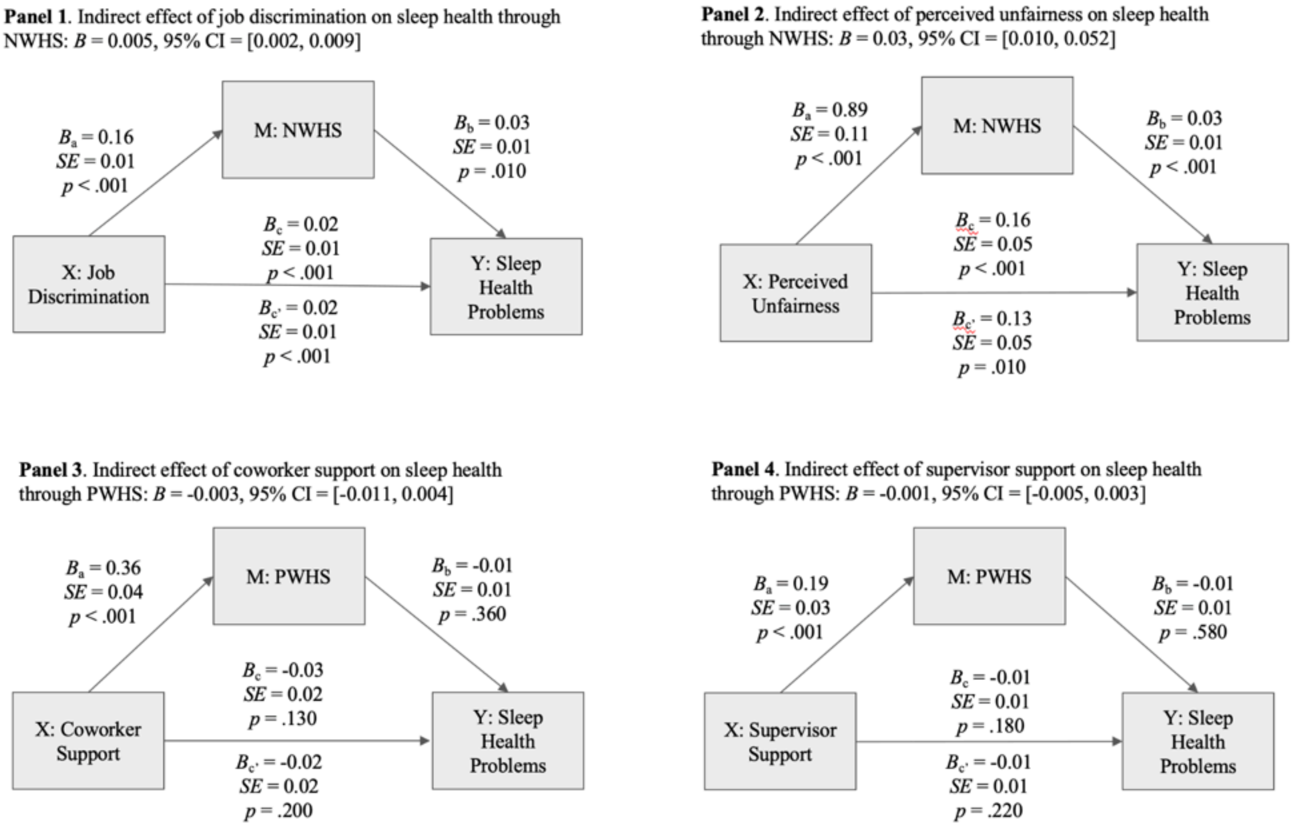

**Figure 3**

*Prospective relationships between work characteristics at T1 and overall sleep health at T2 mediated by work-to-home spillover from T1.*

### Supplementary Files

This is a list of supplementary files associated with this preprint. Click to download.

- SupplementalTables.docx
